# *Moss-derived* recombinant Factor H, CPV-104, effectively antagonizes alternative pathway C3/C5 convertases stabilization by NeFs from patients with primary C3 glomerulopathy

**DOI:** 10.3389/fimmu.2026.1860823

**Published:** 2026-07-01

**Authors:** Zahra Imanifard, Francesca Penati, Sofia Padoa, Paulina Dabrowska-Schlepp, Elena Bresin, Tobia Peracchi, Ariela Benigni, Giuseppe Remuzzi, Marina Noris, Roberta Donadelli

**Affiliations:** 1 Istituto di Ricerche Farmacologiche Mario Negri IRCCS, Clinical Research Center for Rare Diseases Aldo e Cele Daccò and Centro Anna Maria Astori, Science and Technology Park Kilometro Rosso, Bergamo, Italy; 2 Department of Nonclinical Development, Eleva GmbH, Freiburg, Germany

**Keywords:** C3 glomerulopathy (C3G), alternative pathway C3/C5 convertases, Factor H (FH), C3 nephritic factor (C3NeF), C5 nephritic factor (C5NeF), CPV-104

## Abstract

**Background:**

C3 glomerulopathy (C3G) is a rare kidney disease caused by uncontrolled activation of the complement alternative pathway (AP), frequently driven by nephritic factors (NeFs) that stabilize the AP C3 and C5 convertases. NeFs are detected in up to 80% of patients and exhibit heterogeneous stabilizing activity, contributing to variable degrees of complement dysregulation. Current complement−targeted therapies reduce complement activation but may compromise immune surveillance by blocking key components of the pathway. Supplementation with the physiological AP regulator factor H (FH) represents an alternative strategy aimed at restoring complement homeostasis while preserving essential immune functions. CPV−104 is a fully functional recombinant human FH produced in moss, with optimized glycosylation and improved pharmacokinetics, offering a promising therapeutic approach for C3G.

**Objectives and Methods:**

We evaluated the ability of CPV−104 to antagonize NeF−mediated stabilization of the AP C3 convertase (C3bBb) in eight C3G patients enrolled in the Italian MPGN/C3G Registry. Patients were classified as C3NeF+ or C5NeF+ based on properdin dependence in convertase stabilization assays. NeF activity was analyzed using solid−phase assays in which C3bBb decay and formation were assessed in the presence of CPV−104 or serum−derived FH (sd−FH). The impact of CPV−104 ex vivo was tested in fluid−phase assays using patient sera, with Ba (convertase formation) and C3a (convertase activity) measured by ELISA.

**Results:**

CPV−104 accelerated decay of NeF−stabilized C3bBb with efficacy comparable to sd−FH. Convertases stabilized by six of eight NeFs were fully dissociated by CPV−104, while two highly potent C3NeFs were partially antagonized. When added during convertase assembly, CPV−104 significantly reduced C3bBb formation in all patients and showed a stronger inhibitory effect than sd−FH. In fluid−phase assays, using patient sera, CPV−104 markedly decreased Ba and C3a generation, with mean inhibition of 68.9% and 51.8%, respectively, and consistently outperformed sd−FH across patient samples.

**Conclusions:**

CPV−104 effectively limits both stabilization and formation of AP C3 convertases in NeF−positive C3G, demonstrating superior functional activity compared with sd−FH. By restoring physiological AP regulation rather than broadly inhibiting complement activation, CPV−104 represents a promising and potentially safer therapeutic strategy for NeF−driven C3G and warrants further clinical investigation.

## Introduction

C3 glomerulopathy (C3G) is a rare kidney disease associated with uncontrolled activation of the complement alternative pathway (AP), leading to deposition of C3 and its cleavage products in glomeruli (1–3). Clinical presentation ranges from asymptomatic hematuria and proteinuria to nephrotic or nephritic syndrome with severe hypertension. Prognosis is poor, with 30%–50% of patients progressing to end-stage renal disease within 10–15 years of diagnosis (1).

C3G is primarily associated with acquired abnormalities of the complement alternative pathway. These include nephritic factors (NeFs) (4), as well as other autoantibodies targeting complement components such as factor B (5, 6) or factor H (7, 8), all of which may contribute to complement dysregulation. NeFs are identified in 50-80% of patients. They represent a heterogeneous group of autoantibodies that stabilize the AP C3 convertase (C3NeF) in both fluid and membrane-bound forms, or stabilize both the AP C3 and C5 convertases (C5NeF) (4, 9–11).

The extent of NeF activity and their different capacity to stabilize either C3 convertase alone or both C3 and C5 convertases, result in varying levels of complement activation, leading to distinct complement profiles (4).

Several complement inhibitors targeting the terminal pathway or the AP C3 convertase (C3bBb), or preventing C3 activation have been tested in clinical trials in C3G patients, with variable clinical response (12). To date, two disease-specific therapies have been approved by the FDA and EMA for C3G: iptacopan (Fabhalta, LNP023), an oral factor B inhibitor, and pegcetacoplan (Empaveli), a subcutaneous C3/C3b inhibitor.

Factor H (FH) is the central physiological regulator of the AP (13, 14), and its supplementation may offer a therapeutic strategy that aligns more closely with the complement system’s natural regulatory mechanisms. Unlike complement inhibitors that broadly block pathway activation—and may therefore interfere with essential immune functions—FH works by restoring balanced control of the AP. By preventing the assembly of the C3bBb convertase and accelerating its natural dissociation, FH supplementation has the potential to re-establish complement homeostasis in C3G while preserving the system’s critical protective and immune-surveillance activities.

Alongside FH supplementation, several engineered complement regulators have been developed with the aim to enhance inhibitory potency and/or improve tissue targeting, including soluble CR1 (sCR1/TP10) (15), miniFH (16, 17), CR2/FH (18), CRIg/FH (19, 20) and the recently described CSL040 (21). Systemic treatment with Factor H has long been considered impractical due to the large doses required and the high production costs. CPV-104, a fully functional recombinant human FH produced in moss, features optimized glycosylation that improves its pharmacokinetics, bioavailability, and therapeutic efficacy, making clinical application a viable prospect (22).

In this study, we aimed to investigate the ability of CPV-104 to antagonize the stabilizing activity of NeFs from C3G patients on AP C3 convertase, compared to serum-derived FH (sd-FH).

We first examined whether CPV-104 could accelerate the decay of NeF-stabilized C3bBb complexes pre-assembled on C3b-coated wells, given that NeFs prevent C3bBb dissociation. We then assessed the ability of CPV-104 to interfere with the formation of new C3bBb complexes, thereby counteracting NeF-mediated effects on convertase assembly. Finally, we analyzed the effects of CPV-104 *ex vivo* in a fluid phase assay using serum from NeF positive C3G patients, measuring Ba and C3a release as indicators of AP C3 convertase formation and activity, respectively.

## Methods

### Study participants

Patients were recruited through the Italian Registry of MPGN, a network established in 2006 under the coordinator of the Clinical Research Centre of Rare Diseases *Aldo e Cele Daccò* at Mario Negri Institute.

Eight patients with a diagnosis of primary C3G were included in this study. According to current guidelines (3), patients with “dominant C3” glomerular staining on immunofluorescence were diagnosed with C3G and further classified by electron microscopy as having either dense deposit disease (DDD) or C3 glomerulonephritis (C3GN) based on the presence or the absence of highly electron dense deposits in the glomerular basement membrane (3). Patients were selected based on the presence of nephritic factors, and were classified as C3NeF (n=4) or C5NeF (n=4) according to the properdin dependence of alternative pathway C3 convertase stabilization, assessed by spontaneous convertase stabilization assays performed in the absence (sCSA) or in the presence (sPCSA) of properdin (11). Two healthy subjects with no history of renal disease were included as controls. All participants provided informed consent to join the ‘Mario Negri’ Biobank for Rare Diseases and Kidney Diseases. The main clinical and biochemical characteristics of the patients are shown in the **Table 1**.

**Table 1:** Patient clinical and biochemical features at the time of serum collection.

# Pt	Diagnosis	Age at onset (yr)	NeFs	sPCSA	sCSA	C3 (mg/dl)	C4 (mg/dl)	sC5b-9 (ng/ml)	Uprot (g/24h)	SCr (mg/dl)	FBAAs	FHAAs
P1	DDD	22	C3NeF	92	100	12	12	215	8.0	3.89	neg	neg
P2	DDD	9.5	C3NeF	92	73	26	8	302	1.25	0.55	neg	neg
P3	C3GN	30	C3NeF	72	45	23	8	1563	0.40	0.60	neg	neg
P4	C3GN	34	C3NeF	84	65	61	14	246	3.0	1.99	neg	neg
P5	C3GN	32	C5NeF	70	33	65	18	191	< 0.3	0.77	neg	neg
P6	C3GN	8	C5NeF	53	33	67	17	109	< 0.3	0.21	neg	neg
P7	C3GN	17	C5NeF	65	30	18	10	3958	8.0	1.06	neg	neg
P8	DDD	21	C5NeF	72	19	29	18	3328	5.0	0.73	pos	neg

Limit of normal range: DDD, dense deposit disease; C3GN, C3 glomerulonephritis; NeFs, nephritic factors; C3NeF, C3NeF properdin-independent; C5NeF, C5NeF properdin-dependent; sPCSA and sCSA, C3 convertase stabilizing activity against spontaneous decay in the presence and in the absence of properdin, respectively; FBAAs, anti-FB autoantibodies; FHAAs, anti-FH autoantibodies. Normal values: sPCSA ≤42%; sCSA ≤37%; serum C3 levels: 79–152 mg/dl; serum C4 levels: 10–40 mg/dl; plasma sC5b-9; <335 ng/ml; Uprot, urinary proteins: < 0.3 g/24 h; serum creatinin, SCr < 0.9 g/24.

The samples used for the research were stored at the Mario Negri Institute Biological Resources Center, in the Biobank for Rare Diseases and Kidney Diseases (Ranica, Bergamo).

### Complement component assays

Complement C3 and C4 levels in serum were measured by kinetic nephelometry. The normal range for C3 is 79–152 mg/dl and C4 is 10–40 mg/dl. SC5b-9 levels were evaluated in EDTA plasma using MicroVue sC5b-9 Plus EIA (SC5b-9 Plus; Quidel). Normal sC5b-9 levels are defined as <335 ng/ml (mean ± 2 SD of 40 healthy control subjects). The presence of anti-FH and anti-FB autoantibodies were evaluated by an Enzyme-Linked ImmunoSorbent Assay (ELISA) (6, 7).

### Materials and IgG purification

Complement proteins (C3b, FB, FD, Properdin, and FH) were obtained from Complement Technologies (Texas, USA). CPV-104 was kindly provided by ELEVA GmbH (Freiburg, Germany).

Patient IgG was purified using the Melon Gel IgG Purification Kit (Thermo Scientific, VWR International PBI srl, Milan, Italy), and subsequently used to test NeFs activity.

### Microplate/western blot NeF assay

The assay was performed as previously described (11). Briefly, microplate wells were coated with 3 μg/ml C3b in PBS1X and incubated overnight at 4 °C. Following washes, C3b-coated wells were incubated for 12 minutes at 25 °C with 1,000 ng/ml FB, 10 ng/ml FD, 500 ng/ml properdin, and 200 μg/ml IgGs purified from patients or controls, in assay buffer (8.1 mM Na_2_HPO_4_, 1.8 mM NaH_2_PO_4_, 0.1% Tween 20, 75 mM NaCl, 0.5% BSA and 10 mM MgCl_2_). Reactions were carried out either in the presence of 2.64 μg/ml sd-FH or CPV-104, corresponding to the physiological molar ratio of FH to FB (baseline + regulators), or with their buffer (PBS, no regulator). Subsequently, the C3bBb complexes were detached from wells with 10 mM EDTA and 1% SDS and subjected to Western blot analyses. To assess spontaneous and regulator-mediated decay, in additional wells, after washing, the C3bBb complexes formed in the absence of regulators were further incubated for 32 minutes at 25 °C with assay buffer alone (spontaneous decay, –), while complexes formed either in the absence or presence of CPV-104 or sd-FH were incubated with buffer containing 2.64, 3.96, 5.28 µg/ml CPV-104 or sd-FH (regulator-mediated decay, +). Following washing, the remaining C3bBb complexes were detached as above, and analyzed by Western blot.

### Western blotting analysis

C3bBb complexes were analyzed by Western blot as previously described (11). Briefly, proteins were separated by SDS-PAGE, transferred to PVDF membranes, and the Bb fragment, corresponding to the active component of the alternative pathway C3 convertase, was detected using a polyclonal goat anti-human FB antibody (Quidel; 1:10,000), followed by an HRP-conjugated anti-goat secondary antibody (Sigma-Aldrich; 1:10,000) and chemiluminescence detection.

To assess the effect of CPV-104 or sd-FH on decay of C3bBb complexes formed in the presence of patient or control IgGs, the percentage of residual Bb band was calculated as the ratio of the intensity (in Pixel^2^) of each Bb band after the decay and the intensity of the corresponding baseline Bb band before decay multiplied by 100.

The threshold for positivity was set as a residual C3 convertase exceeding the upper limit of the normal range (mean + 2 SD of values obtained from control IgG samples, (n=30). Specifically, in the presence of properdin, thresholds were > 42% for spontaneous decay (sPCSA) and > 10% for FH-mediated decay (FHPCSA). In the absence of properdin, thresholds were > 37% for spontaneous decay (sCSA) and > 12% for FH-mediated decay (FHCSA) (11).

To evaluate the effect of CPV-104 and sd-FH on C3 convertase formation, we calculated the ratio of the intensity (in Pixel^2^) of the Bb band from the reaction with sd-FH or CPV-104 (baseline + regulator) and the intensity of the Bb band from the corresponding reaction in their absence (baseline, -), multiplied by 100.

When CPV-104 or sd-FH was added during both the formation or decay phases, the percentage of residual Bb was determined as the ratio of Bb band intensity (in Pixel²) after decay in the reaction with either regulator to the baseline Bb band intensity prior to decay in the reaction without regulators multiplied by 100. Band intensities were quantified by densitometry using NIH Software ImageJ (NIH, USA).

### Fluid phase assay

Fluid-phase experiments were performed by incubating 20% serum from patients with C3G or from healthy subjects, diluted in PBS containing 10 mM MgCl₂. The incubation was performed on 3 μg/ml C3b-coated wells at 30 °C for 30 minutes (T1) in the presence of 40 μg/ml CPV-104 or sd-FH, or with their buffer (PBS, no regulator). CPV-104 and sd-FH were used at 40 μg/ml, corresponding approximately to the FH concentration expected in 20% serum at the lower end of the physiological reference range. C3a levels, marker of C3 convertase activity, and Ba levels, marker of C3 convertase formation, were quantified by ELISA at baseline (T0) and after incubation (T1). To determine the complement activation occurring during the incubation period, baseline levels measured at T0 were subtracted from the corresponding values measured at T1. The levels of C3a and Ba generated in the presence of CPV-104 or sd-FH were compared with those obtained in the absence of regulators. The percentage of inhibition calculated as {1- [(T1 + regulator – T0)/(T1 No regulator – T0)]} × 100.

### Statistical analysis

For each of 8 subjects, 2 or 3 replicates were performed; all analyses were conducted on per-subject mean values. Data are presented as mean ± SEM. In the solid-phase experiment, the primary endpoint was the inhibitory effect of CPV-104 on C3bBb stabilization by NeFs, assessed across three concentrations (2.64, 3.96, 5.28 µg/ml) by comparing residual C3bBb after CPV-104-mediated decay to spontaneous decay, and by comparing C3bBb at baseline (no decay phase) against the reference value of no inhibition. In the fluid-phase experiment, the primary endpoint was the reduction in AP C3 convertase formation and activity in the presence of CPV-104, assessed by comparing Ba and C3a generation to the no regulator condition. Prior to hypothesis testing, normality of each variable was assessed by visual inspection of Q-Q plots and the Shapiro-Wilk test. In the solid-phase experiment, CPV-104 efficacy at each concentration (2.64, 3.96, 5.28 µg/ml) was assessed by paired t-test against spontaneous decay; baseline C3bBb levels were compared to the reference value of no inhibition by one-sample t-test (μ = 100%). In the fluid-phase experiment, Ba and C3a generation in the presence of CPV-104 were compared to the no regulator condition by paired t-test. All secondary comparisons were performed using paired t-tests or one-sample t-tests, as appropriate. To control for multiple comparisons, a Bonferroni correction was applied separately within each experimental phase: α = 0.0125 for the four solid-phase primary tests and α = 0.025 for the two fluid-phase primary tests; all secondary comparisons were evaluated at the nominal α = 0.05. All statistical analyses were performed using SAS version 9.4 (SAS Institute Inc., Cary, NC, USA). Figures were generated using R (version 4.5.2; R Foundation for Statistical Computing, Vienna, Austria), RStudio (version 2026.01.0; Posit Software, Boston, MA, USA), and Microsoft Excel (Microsoft Corp., Redmond, WA, USA).

## Results

### Patients

Eight patients with C3G and NeF positivity were included in this study (**Table 1**); three were diagnosed with dense deposit disease (DDD; P1, P2, P8) and five with C3 glomerulonephritis (C3GN; P3-P7). Age at disease onset ranged from 8 to 34 years. All clinical and laboratory parameters reported in **Table 1** refer to the time of sample collection. None of the patients were receiving complement inhibitors at that time. Based on the properdin dependence of AP C3 convertase stabilization -assessed by spontaneous convertase stabilization assays performed in the presence (sPCSA) or absence (sCSA) of properdin- patients were classified as C3NeF^+^ (properdin-independent, n=4; P1-P4) or C5NeF^+^ (properdin-dependent, n=4; P5-P8).

Serum C3 levels were lower than normal in all patients, whereas C4 levels were normal or close to the lower limit of the normal range (**Table 1**). Plasma sC5b-9 levels were markedly elevated in three of eight patients (P3, P7, and P8) (**Table 1**). Proteinuria was present in all patients except P5 and P6, with nephrotic-range values (≥3.5 g/24h) observed in P1, P7 and P8 (**Table 1**). Elevated serum creatinine levels were observed in three patients (P1, P4, and P7) (**Table 1**). Anti–factor B antibodies were detected in one patient (P8), whereas anti–factor H antibodies were negative in all tested patients (**Table 1**).

### Effect of CPV-104 on decay of NeF-stabilized AP C3 convertases

**Figure 1:**
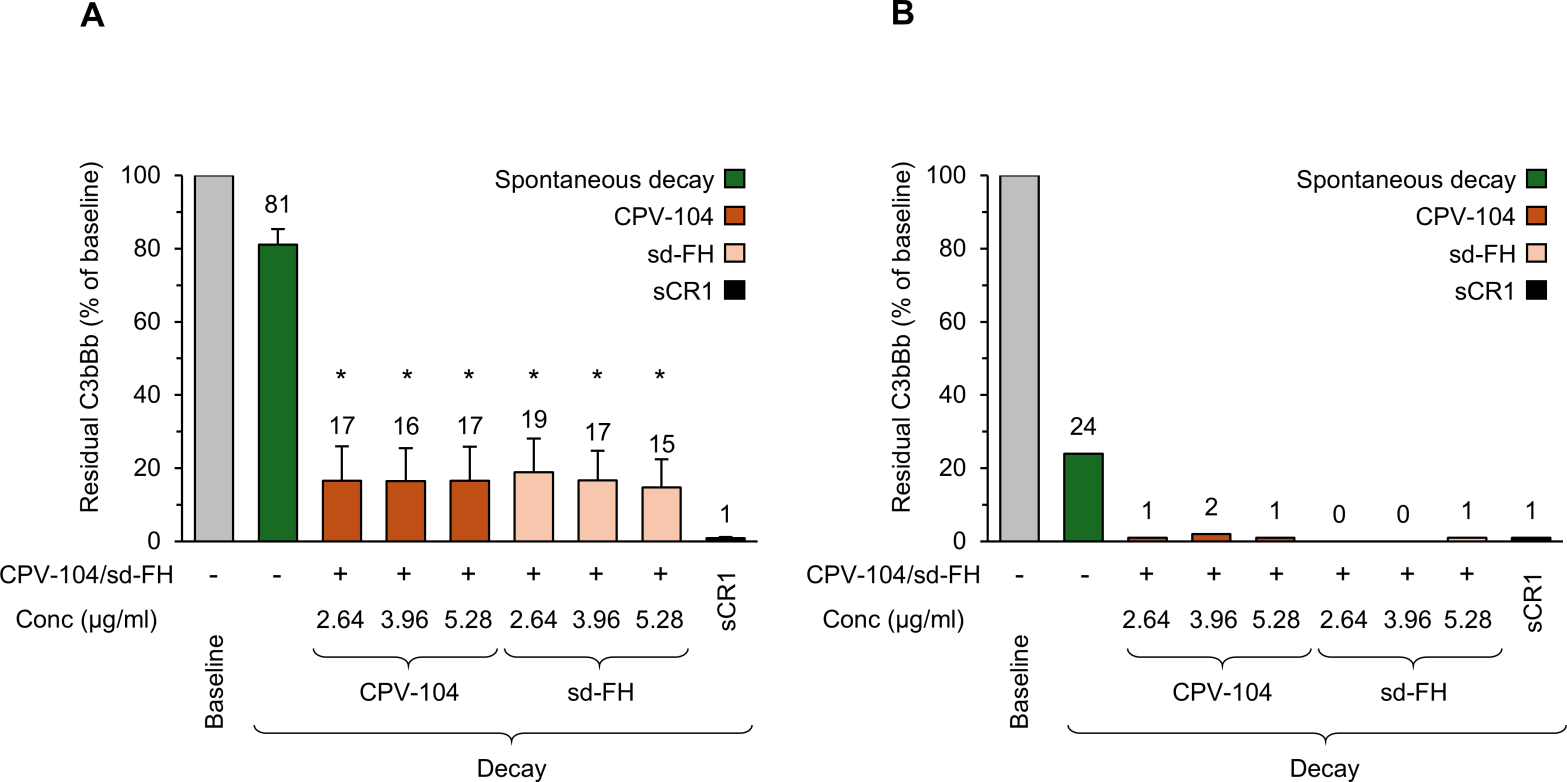
Effect of CPV-104 on decay of NeF-stabilized C3bBb in a microplate/Western blot (WB) assay. **(A)** Spontaneous and CPV-104 or sd-FH-mediated decay of AP C3 convertase formed in the presence of NeFs isolated from eight C3G patients. After AP C3 convertase assembly, decay of C3bBb complexes was assessed by incubation with buffer alone (spontaneous decay, –) or buffer containing 2.64, 3.96, and 5.28 µg/ml CPV-104 or sd-FH (regulator-mediated decay, +). The percentage of residual C3bBb was calculated as the ratio between the densities of each Bb band after decay and the corresponding baseline Bb band before decay x 100. Results are expressed as mean ± SEM of the experiments in the eight patients. *P < 0.001 vs. spontaneous decay. **(B)** Decay of AP C3 convertase in the presence of IgGs from healthy controls (n = 2), assessed under the same experimental conditions as in **(A)**. Data are expressed as mean of 2 experiments.

The ability of CPV-104 to counteract the stabilizing activity of NeFs from 8 C3G patients (4 C3NeF^+^ and 4 C5NeF^+^, **Table 1**) on AP C3 convertase decay was evaluated by adding CPV-104 or sd-FH at three concentrations (2.64, 3.96, 5.28 µg/ml) during the decay step. The lowest concentration was selected to achieve a 2:1 molar ratio of FH with FB added during convertase assembly phase, close to ratio in normal plasma. Both CPV-104 and sd-FH were equally effective in accelerating decay in the presence of NeFs, already at the lowest dose (*P* < 0.001, vs spontaneous decay **Figure 1A**), without a dose-dependent effect.

However, the response varied among patients. Both CPV-104 and sd-FH led to the completedissociation of C3bBb complexes stabilized by NeFs from six out of eight patients (P3-P8) with no detectable residual C3bBb complexes observed after the decay step (**Supplementary Table 1**, **Supplementary Figure 1A**). At variance, the NeFs (C3NeFs) from patients P1 and P2 were only partially antagonized byCPV-104 and sd-FH at all doses, resulting in incomplete dissociation of C3bBb complexes (**Supplementary Table 1**, **Supplementary Figure 1B**). Of note, P1 and P2 NeFs showed the highest sPCSA and sCSA values (**Table 1**). As expected, both CPV-104 and sd-FH fully dissociated the C3bBb complexes in the presence of control IgG (**Figure 1B**).

### Effect of CPV-104 on formation of NeF-stabilized AP C3 convertase

**Figure 2:**
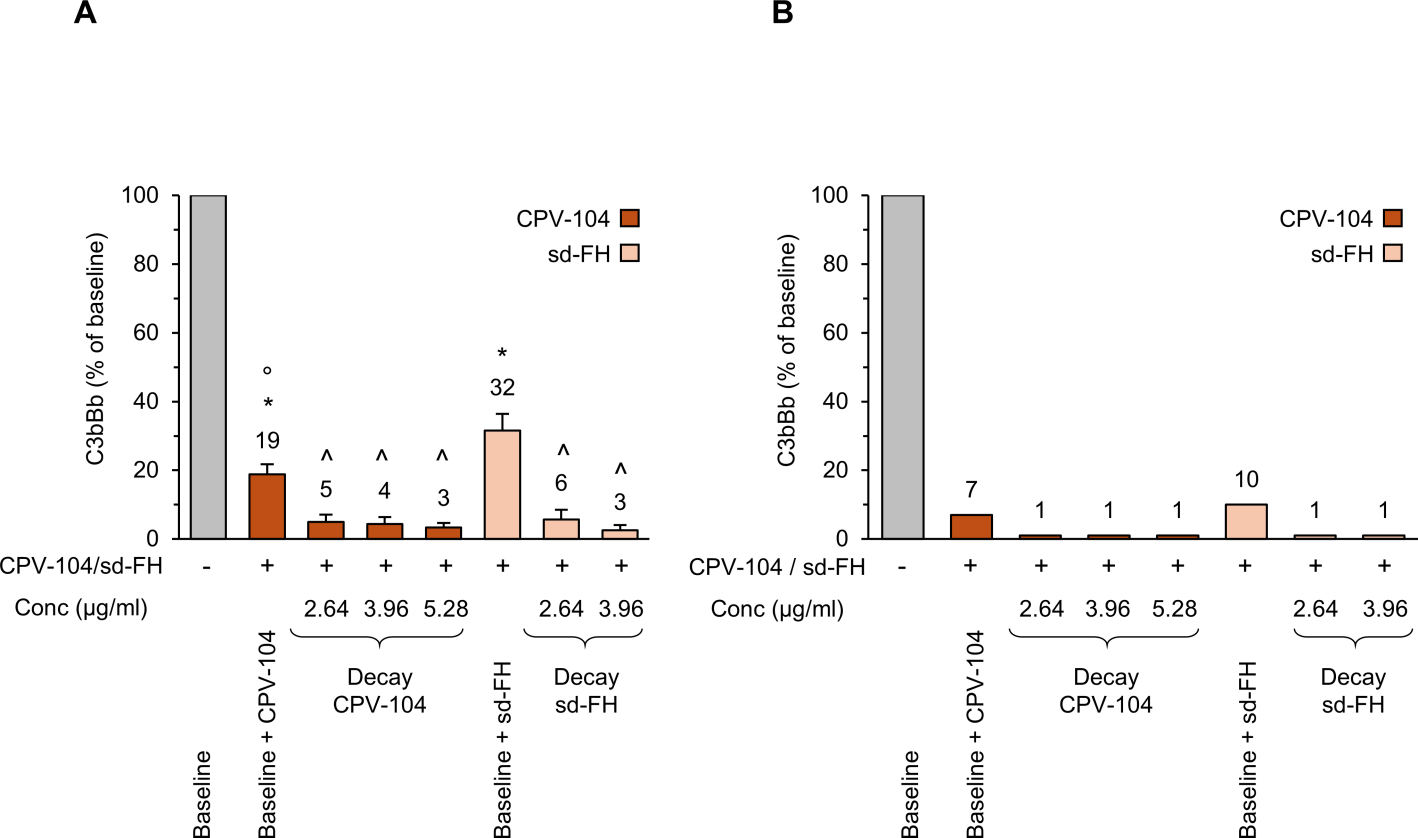
Effect of CPV-104 on formation and decay of NeF-stabilized C3bBb in a microplate/Western blot (WB) assay. **(A)** Effect of CPV-104 on the formation and subsequent decay of AP C3 convertase in the presence of nephritic factors (NeFs) from eight C3G patients. C3 convertase complexes were formed either in the absence (baseline -) or presence of 2.64 µg/ml of CPV-104 (baseline + CPV-104) or sd-FH (baseline + sd-FH). The C3bBb complexes formed in the presence of CPV or sd-FH, were subsequently allowed to decay with buffer containing CPV-104 (2.64, 3.96, or 5.28 µg/ml) or sd-FH (2.64 or 3.96 µg/ml). The percentage of C3bBb was calculated as the ratio of the densities of each Bb band -either after formation in the presence of regulators or following decay- and the baseline Bb band before decay in the absence of regulators (baseline -), x 100. Results are expressed as mean ± SEM of the experiments in the eight patients. *P < 0.001 vs. baseline, °*P* < 0.001 vs. baseline + sd-FH, ^ *P* < 0.001 vs. corresponding baselines (plus CPV-104 or sd-FH). **(B)** Effect of CPV-104 on C3bBb formation and decay in the presence of IgGs from healthy controls (n = 2), assessed under the same experimental conditions as in **(A)**.

The effect of CPV-104 on the formation of C3bBb in the presence of NeFs was investigated by adding 2.64 µg/ml CPV-104 during the convertase assembly phase (baseline + regulator). Compared with reactions performed in the absence of regulators (baseline, -), CPV-104 significantly reduced C3bBb formation in the presence of NeFs from all eight patients (*P* < 0.001, **Figure 2A**; **Supplementary Table 2**), including P1 and P2, whose NeFs showed the strongest stabilizing activity on C3bBb spontaneous decay (**Table 1**) and were resistant to FH-mediated decay (**Supplementary Table 1**, **Supplementary Figure 1B**). Of relevance, CPV-104 exerted a significantly stronger inhibitory effect on C3 convertase formation than sd-FH (*P* < 0.001, **Figure 2A**).

When either CPV-104 or sd-FH was added both during the assembly and the decay phases, C3bBb complexes were further reduced after decay in all patients compared with their corresponding baselines measured at the end of assembly (*P* < 0.001, **Figure 2A**; **Supplementary Table 2**).

Namely, no or very faint C3bBb was detected after decay in the presence of NeFs from six out of eight patients (P3-P8) with all tested concentrations of CPV-104 and sd-FH (**Supplementary Table 2**, **Supplementary Figure 2A**). In the presence of NeFs from P1 and P2, C3bBb was still detectable at the end of thereaction, however values were one quarter of those observed with the CPV-104 added only during thedecay phase (**Supplementary Tables 1**–**2**, **Supplementary Figure 2B**). As expected, both CPV-104 and sd-FH almost completely prevented C3bBb formation in the presence of control IgG (**Figure 2B**).

### Effect of CPV-104 on C3 convertase formation and activity by NeF ex vivo

To determine whether the inhibitory activity of CPV-104 observed in the *in vitro* solid-phase assays also translated into reduced C3 convertase formation and activity in NeF^+^ sera, we next evaluated its effect in fluid phase assays using sera from the same eight C3G patients.

C3 convertase activity was assessed by measuring the release of C3a, whereas Ba was used as a marker of convertase formation.

**Figure 3:**
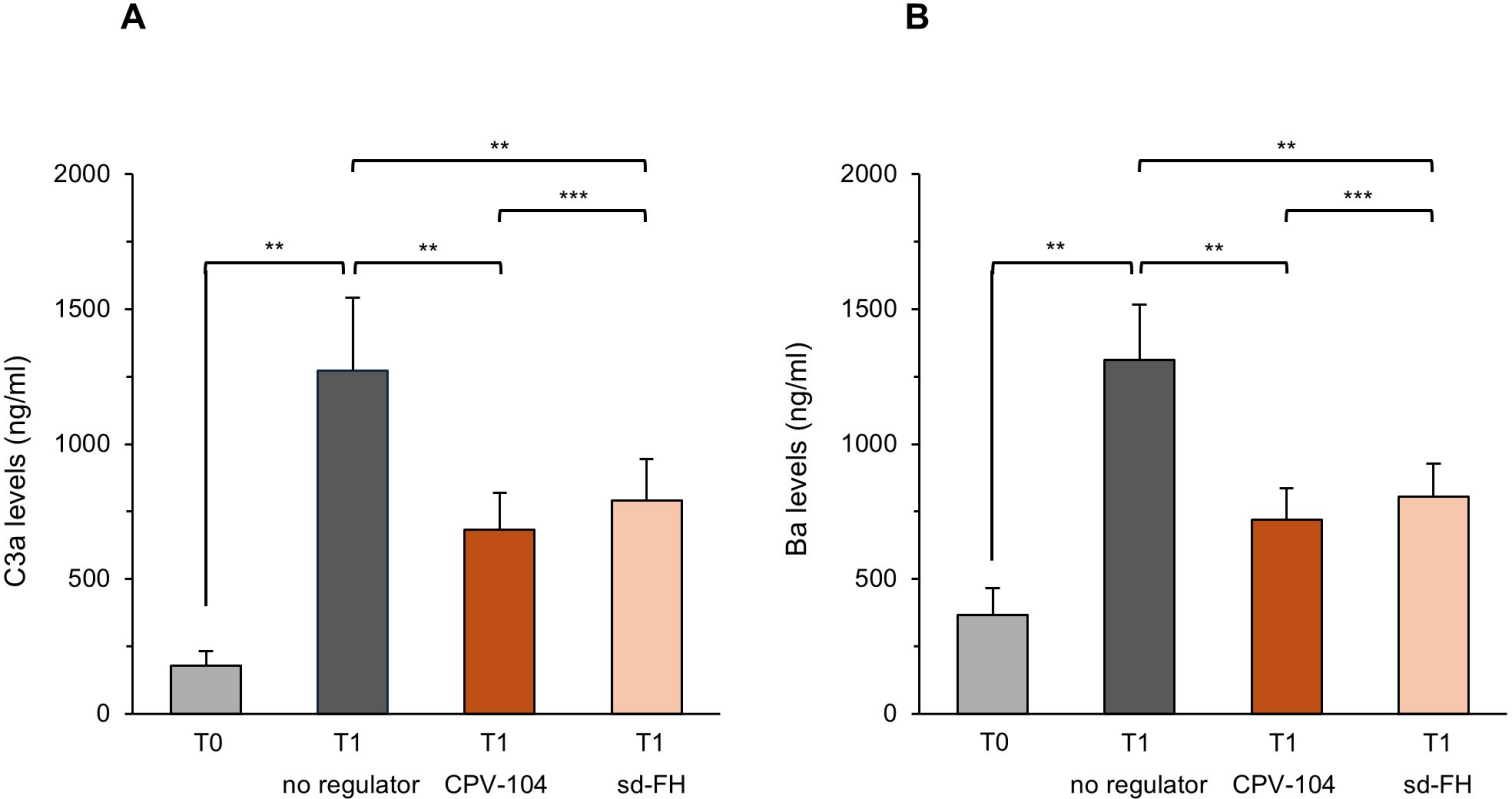
Effect of CPV-104 on complement activation in fluid phase. **(A–B)** C3 convertase activity and formation in sera from eight NeF positive C3G patients were assessed by measuring C3a **(A)** and Ba **(B)** release at baseline (T0) and after incubation (T1) in the absence (no regulator) or presence of 40 μg/ml CPV-104 or sd-FH. Bars represent mean ± SEM (n = 8). Statistical significance was assessed using paired t-test. ***P < 0.05; **P < 0.01.

In the absence of exogenous regulators, incubation of patient sera resulted in significant complement activation, with C3a levels increasing from 179.1 ± 54.8 ng/ml at baseline (T0) to 1272.6 ± 268.8 ng/ml after incubation (T1) (*P* = 0.003), and Ba levels increasing from 368.1 ± 97.6 ng/ml (T0) to 1311.3 ± 205.5 ng/ml (T1) (*P* = 0.004) (mean ± SEM; n = 8) (**Figures 3A, B**).

Addition of CPV-104 significantly reduced complement activation markers (C3a: *P =* 0.004 vs no regulator; Ba: *P =* 0.001 vs no regulator) (**Figures 3A, B**).

Similar results were obtained when T0 values were subtracted as blanks and data were expressed as Δ values (T1–T0), with a mean inhibition of 51.8 ± 5.3% for C3a and 68.9 ± 7.3% for Ba. Accordingly, C3a levels decreased from 1094.0 ± 240.6 ng/ml to 504.6 ± 107.1 ng/ml and Ba levels from 943.2 ± 220.4 ng/ml to 350.3 ± 119.8 ng/ml (**Figures 3A, B**).

**Figure 4:**
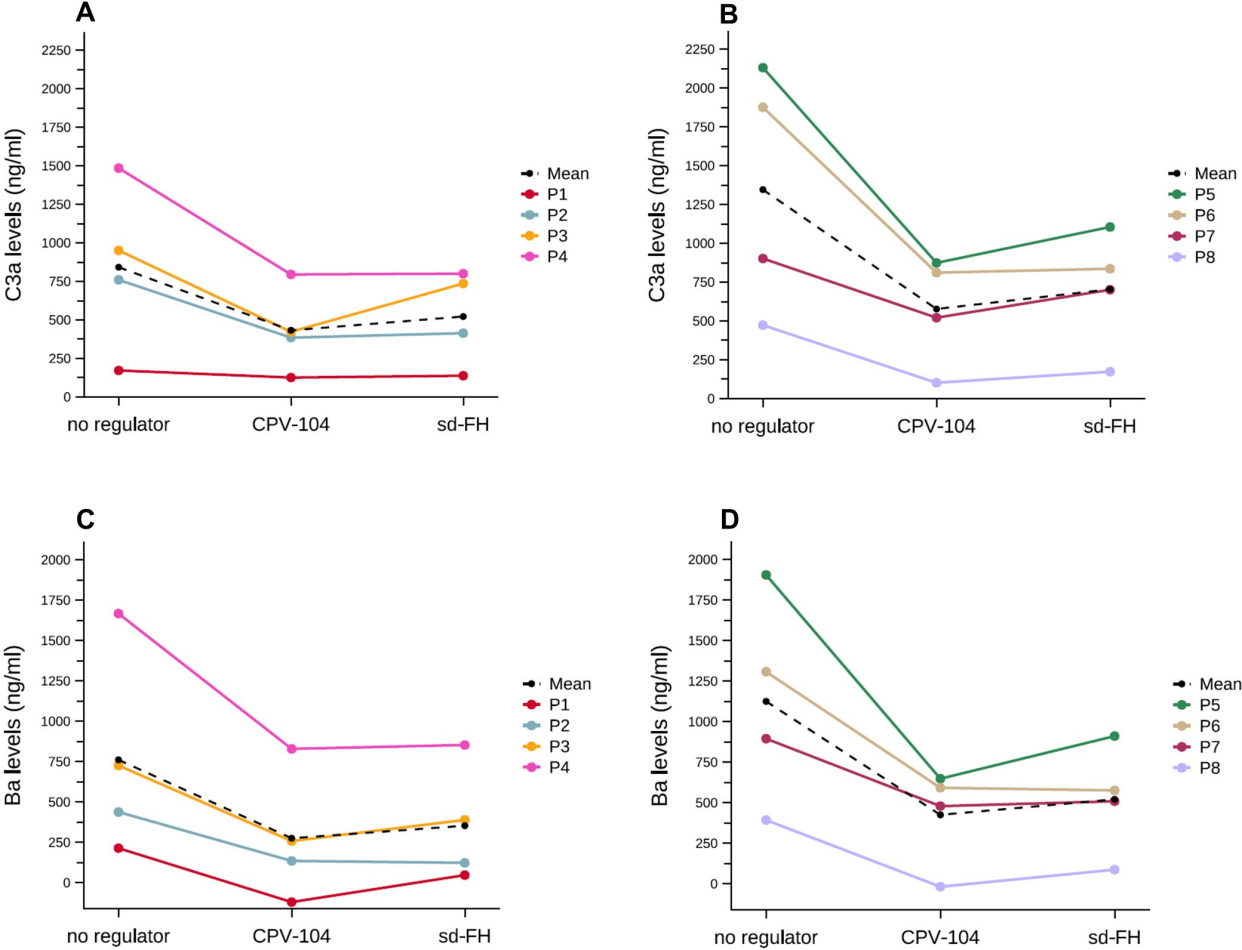
Individual response to CPV-104 and sd-FH in fluid-phase complement activation. **(A–D)** C3 convertase activity and formation in sera from eight NeF positive C3G patients were assessed by measuring C3a and Ba release in the absence (no regulator) or presence of 40 μg/ml CPV-104 or sd-FH. C3a release is shown for C3NeF-positive patients (panel A) and C5NeF-positive patients (panel B). Ba release is shown for C3NeF-positive patients (panel C) and C5NeF-positive patients (panel D). Values are expressed as Δ values (T1–T0; ng/ml), obtained by subtracting baseline levels (T0) from post-incubation levels (T1). Each line represents an individual patient (P1–P8), and the black dashed line indicates the mean value within each group.

Analysis of individual patient responses, shown separately for C3NeF-positive and C5NeF-positive patients, demonstrated that CPV-104 reduced both C3a and Ba release in all eight sera, although the magnitude of inhibition varied among patients (**Figures 4A–D**).

By comparison, sd-FH also reduced complement activation across all eight patient sera, with mean C3a and Ba inhibition of 40.4 ± 5.9% and 59.3 ± 5.1%, respectively. Direct comparison between the two regulators showed that CPV-104 exerted a significantly stronger inhibitory effect than sd-FH on both C3a (*P* = 0.022) and Ba release (*P* = 0.036) (**Figures 3A, B**).

**Figure 5:**
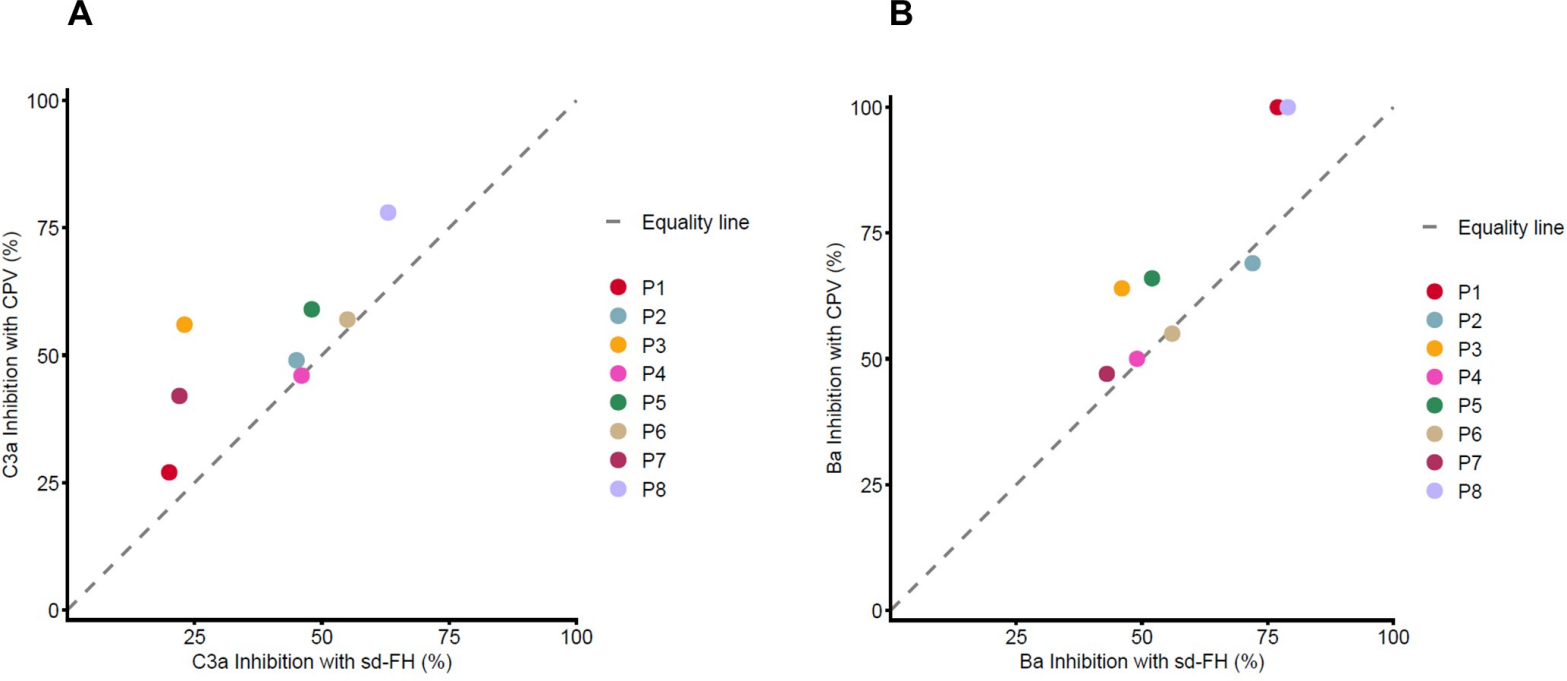
Comparison of CPV-104 and sd-FH inhibitory activity on complement activation. Equality plots showing the percentages of inhibition of C3a **(A)** and Ba **(B)** release achieved with CPV-104 (y-axis) versus those with sd-FH (x-axis) in sera from eight C3G patients. Each point represents an individual patient (P1–P8). Percent inhibition was calculated based on Δ(T1–T0) values. The dashed line indicates the line of identity (y = x). Data points above the line indicate greater inhibition by CPV-104 compared with sd-FH.

Consistently, equality plots comparing percent inhibition achieved by CPV-104 and sd-FH showed most data points lying above the equality line (C3a: 7/8; Ba: 5/8), indicating overall stronger inhibition with CPV-104 than sd-FH (**Figures 5A, B**). Across individual patients, the degree of inhibition achieved with CPV-104 significantly correlated with that observed with sd-FH for both C3a (r = 0.76, P = 0.03) and Ba (r = 0.88, *P* = 0.004).

## Discussion

Current complement-targeted therapies effectively reduce complement activation, yet they also present important limitations. By blocking key components of the complement cascade, these drugs may compromise immune surveillance, increase susceptibility to infections, and alter immune homeostasis (12). These limitations highlight the need for alternative strategies that modulate complement activity while preserving essential host-defense functions. In this context several efforts have been made to obtain preparations of the natural complement alternative pathway regulator FH for systemic administration (23–26). However, purification of FH from plasma is constrained by several challenges, including the low recovery yield -insufficient for therapeutic needs- high production costs, and the inherent risk of pathogen transmission. These limitations have driven extensive efforts toward the development of recombinant FH through heterologous expression systems, which however have been hindered by low yields or immunogenicity arising from differences in post-translational modification compared to native FH (24).

Moss bioreactors provide a compelling solution to these limitations. Unlike serum-derived FH, moss based production ensures a pathogen free, fully controlled, and scalable manufacturing process. Moreover, moss enables precise engineering of post-translational modifications, resulting in a homogeneous and functionally consistent recombinant FH preparation with substantially improved batch reproducibility and markedly higher yields (22, 27).

Our findings demonstrate that moss-derived FH, CPV-104, effectively counteracts the stabilizing activity of NeFs on AP C3/C5 convertases, highlighting its potential as a therapeutic agent for C3G. Notably, CPV-104 showed an efficacy comparable to FH derived from human serum in promoting dissociation of NeF-stabilized C3 convertase, confirming that this recombinant molecule retains full regulatory activity.

NeFs are highly heterogeneous in their binding properties and ability to stabilize AP C3/C5 convertases (4, 11), and this heterogeneity was reflected in our findings. Indeed, CPV-104 and sd-FH fully dissociated convertases stabilized by six of the eight NeFs tested (including two C3NeF and four C5NeF), whereas the two C3NeF with highest stabilizing activity were only partially antagonized.

A key finding of our study is that, CPV-104, at physiological concentration, significantly limited the formation of C3bBb, when added during C3bBb assembly in the presence of all NeFs tested. Data that CPV-104 reduced C3bBb formation more effectively than sd-FH likely can be attributed to the fact that sd-FH consists of a heterogeneous mixture of naturally occurring polymorphic variants, whereas CPV-104 is a homogeneous recombinant preparation carrying the Ile62/Tyr402 variants (22). Indeed, the Ile62 variant has been associated with higher C3b-binding affinity and enhanced complement regulatory activity compared with the Val62 variant (22, 27, 28). Consistently, previous data demonstrated that the Ile62/Tyr402 variant of CPV-104 binds to C3b with a higher affinity than both sd-FH and the alternative polymorphic variant (Val62/His402) in C3b ELISA binding assay as well as in SPR analysis (22).

Although FH is well known for accelerating convertase decay, its role in limiting C3bBb formation remains poorly investigated. Traditionally, FH has been proposed to compete with FB for binding to C3b, thereby limiting the assembly of the C3 proconvertase (C3bB). However, our previous study showed that FH does not significantly affect C3bB proconvertase formation, suggesting instead that its inhibitory effect on C3bBb generation may occur by promoting dissociation of nascent C3bBb and/or preventing the conversion of C3bB into C3bBb (11).

Additional studies are required to elucidate the precise mechanism by which CPV-104 interferes with C3bBb formation in the presence of NeFs. We hypothesize that CPV-104 may bind C3b within nascent convertase complexes and interfere with effective NeF engagement, thereby reducing NeF-mediated stabilization of C3bBb. This proposed mechanism could help explain the effective inhibition observed with CPV-104, even against the most potent NeFs.

Importantly, the inhibitory activity of CPV-104 observed in the solid-phase convertase assays *in vitro* was corroborated in fluid phase experiments using serum from the same C3G patients. In this physiologically relevant setting, CPV-104 significantly reduced release of both Ba and C3a, indicating effective suppression of both C3 convertase formation and activity. Again, CPV-104 showed stronger inhibitory effect than sd-FH, consistent with its higher C3b affinity and its ability to restrain *de novo* convertase formation.

While the number of patients included in this study is relatively small -particularly after stratification according to NeF profile- the use of a cohort specifically selected for functionally characterized NeF activity provides a valuable opportunity to explore underlying mechanisms and potential therapies. Although this cohort was not designed to fully represent the broader C3G population, it enables focused insight, and our findings should therefore be viewed as primarily mechanistic and exploratory.

In conclusion, CPV-104 may represent a promising therapeutic candidate for C3G, particularly for NeF-driven disease. CPV-104 does not only accelerate convertase decay but -critically- limits convertase assembly, a dual mechanism that may be advantageous in conditions characterized by persistent convertase stabilization. By restoring physiological AP regulation rather than broadly inhibiting complement activation, CPV-104 might offer a more physiological and potentially safer approach compared with currently available complement inhibitors. The potential advantages of CPV-104 over currently available complement-targeted therapies remain to be confirmed in clinical studies. These findings support further clinical investigation of CPV-104 as a novel therapeutic strategy for C3G.

## Data Availability

The original contributions presented in the study are included in the article/**Supplementary Material**. Further inquiries can be directed to the corresponding author.

## Supplementary material

Supplementary Table 1 Residual C3bBb levels after CPV-104 or sd-FH-mediated decay of NeF-stabilized convertases.

Supplementary Table 2 C3bBb levels after formation in the presence of CPV-104 or sd-FH and subsequent regulator-mediated decay of NeF-stabilized convertases.

Supplementary Figure 1 **(A-B)**; Spontaneous and CPV-104 or sd-FH-mediated decay of C3bBb in the presence of NeFs by microplate/Western blot (WB) assay.

Supplementary Figure 2 **(A-B)**: Effect of CPV-104 and sd-FH on C3bBb formation and decay in the presence of NeFs by microplate/Western blot (WB) assay.
