# Supplementary material for: Moss-derived recombinant Factor H, CPV-104, effectively antagonizes alternative pathway C3/C5 convertases stabilization by NeFs from patients with primary C3 glomerulopathy
Source: Front Immunol. 2026 Jul 1;17:1860823. doi: 10.3389/fimmu.2026.1860823 (PMC13368663; doi:10.3389/fimmu.2026.1860823)
Supplement: Supplementary Table 1 — Residual C3bBb levels after CPV-104 or sd-FH-mediated decay of NeF-stabilized convertases. [file DataSheet1.pdf]

In vitro efficacy of recombinant Factor H, CPV-104, to antagonize alternative pathway C3/C5 convertases stabilization by NeFs from patients with primary C3 glomerulopathy (C3G)

Zahra Imanifard[1], Francesca Penati[1], Sofia Padoa[1], Paulina Dabrowska-Schlepp[2], Elena Bresin[1], Tobia Peracchi[1], Ariela Benigni[1], Giuseppe Remuzzi[1], Marina Noris[1], Roberta Donadelli[1]

Supplementary Material

- Supplementary Table S1; Residual C3bBb levels after CPV-104 or sd-FH-mediated decay of NeF-stabilized convertases.
- Supplementary Table S2; C3bBb levels after formation in the presence of CPV-104 or sd-FH and subsequent regulator-mediated decay of NeF-stabilized convertases.
- Supplementary Figure S1. (A-B); Spontaneous and CPV-104 or sd-FH-mediated decay of C3bBb in the presence of NeFs by microplate/Western blot (WB) assay.
- Supplementary Figure S2. (A-B): Effect of CPV-104 and sd-FH on C3bBb formation and decay in the presence of NeFs by microplate/Western blot (WB) assay.

Supplementary Material

Supplementary Table S1.

Patients	regulator-mediated decay (% of residual C3bBb)					
	CPV-104			sd-FH		
	2.64 μg/ml	3.96 μg/ml	5.28 μg/ml	2.64 μg/ml	3.96 μg/ml	5.28 μg/ml
P1	63	60	61	62	51	52
P2	56	56	58	59	56	48
P3	2	2	2	3	3	2
P4	4	5	4	12	10	8
P5	1	0	0	0	0	0
P6	2	2	2	3	2	2
P7	2	2	3	5	3	3
P8	5	6	4	9	8	5

Supplementary table S1. Residual C3bBb levels after CPV-104 or sd-FH-mediated decay of NeF-stabilized convertases. C3bBb complexes were assembled on C3b-coated wells in the presence of IgGs purified from eight C3G patients (P1–P8) and subsequently allowed to decay in the presence of CPV-104 or sd-FH at the indicated concentrations. Values represent the mean percentage of residual C3bBb for each patient, calculated as the ratio between Bb band intensity after the decay step and the corresponding baseline Bb band before decay ×100. Data are expressed as mean of n = 2–3 independent replicates per patient.

Supplementary Table S2

Patients	C3bBb (% of baseline)		regulator-mediated decay (% of residual C3bBb)				
	baseline plus CPV-104	baseline plus sd-FH	CPV-104			sd-FH	
			2.64 µg/ml	3.96 µg/ml	5.28 µg/ml	2.64 µg/ml	3.96 µg/ml
P1	28	44	15	15	11	22	13
P2	29	49	14	12	7	13	4
P3	9	21	0	0	0	0	0
P4	23	40	4	3	3	5	1
P5	8	15	0	0	0	0	0
P6	12	13	0	0	0	0	0
P7	20	37	3	3	2	2	2
P8	23	35	5	3	4	5	2

Supplementary Table S2. C3bBb levels after formation in the presence of CPV-104 or sd-FH and subsequent regulator-mediated decay of NeF-stabilized convertases. C3bBb complexes were assembled on C3b-coated wells in the presence of IgGs purified from eight C3G patients (P1–P8), either in the absence (baseline) or presence of CPV-104 or sd-FH (2.64 µg/ml). Convertases formed in the presence of CPV-104 or sd-FH were subsequently allowed to decay in the presence of the regulators at the indicated concentrations (regulator- mediated decay). Values represent the mean percentage of C3bBb for each patient, calculated as the ratio between Bb band intensity, either after formation in the presence of regulators or after the decay step, and the corresponding baseline Bb band formed in the absence of regulators ×100. Data are expressed as mean of n = 2 independent replicates per patient.

Supplementary Figures S1 (A-B)

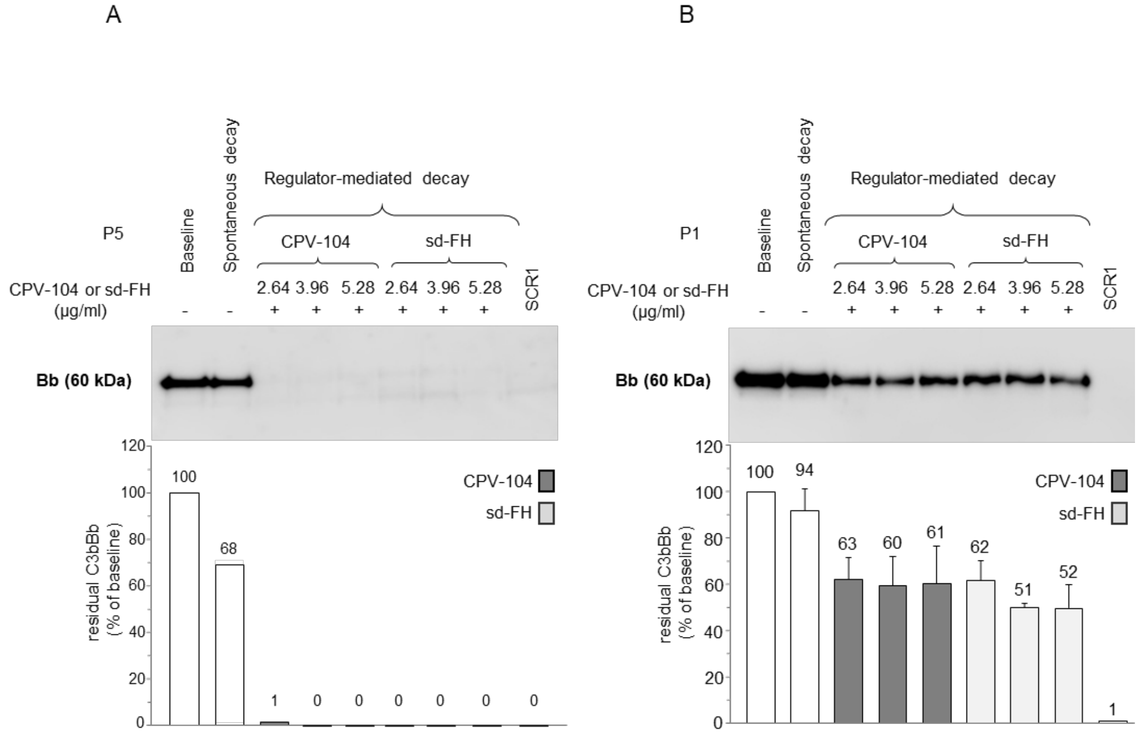

Supplementary Figure S1. Spontaneous and CPV-104 or sd-FH-mediated decay of C3bBb in the presence of NeFs by microplate/Western blot (WB) assay. (A-B) Representative Western blot images and corresponding quantification of spontaneous and CPV-104 or sd-FH-mediated decay of AP C3bBb in the presence of NeFs from patient P5 (A) and P1 (B). C3bBb complexes were assembled by incubating C3b-coated wells with 1,000 ng/ml FB, 10 ng/ml FD, 500 ng/ml properdin, and 200 µg/ml IgGs purified from patients (baseline). In additional wells, after washing, formed complexes were incubated at 25°C for 32 minutes either with buffer alone (spontaneous decay, –) or with buffer containing CPV-104 or sd-FH at concentrations of 2.64, 3.96, and 5.28 µg/ml (regulator-mediated decay, +). Residual C3bBb was expressed as percentage of baseline, calculated as the ratio between Bb band intensity after decay and the corresponding baseline Bb band before decay ×100. Data are shown as mean of n = 2 independent experiments for P5 (A) and as mean ± SD of n = 3 independent experiments for P1 (B).

Supplementary Figures S2 (A-B)

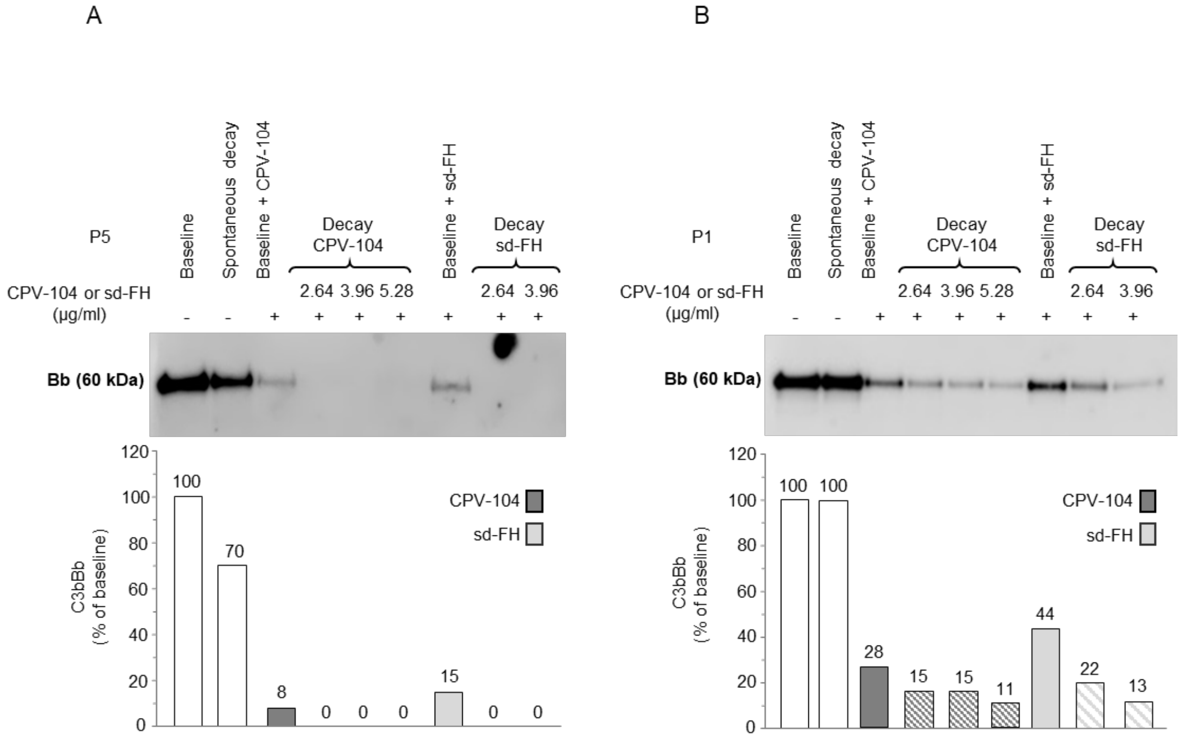

Supplementary Figure S2. Effect of CPV-104 and sd-FH on C3bBb formation and decay in the presence of NeFs by microplate/Western blot (WB) assay. (A–B) Representative Western blot images and corresponding quantification of C3bBb formation and subsequent decay in the presence of nephritic factors (NeFs) from patient P5 (A) and P1 (B). C3bBb complexes were assembled by incubating C3b-coated wells with 1,000 ng/ml FB, 10 ng/ml FD, 500 ng/ml properdin, and 200 µg/ml IgGs purified from patients, either in the absence (-, baseline) or presence of 2.64 µg/ml CPV-104 or sd-FH. In additional wells, after washing, formed complexes were incubated at 25°C for 32 minutes either with buffer alone (spontaneous decay, -). C3bBb formed in the presence of 2.64 µg/ml CPV-104 or sd-FH were incubated with buffer containing CPV-104 (2.64, 3.96, or 5.28 µg/ml) or sd-FH (2.64 or 3.96 µg/ml) (regulator-mediated decay +). C3bBb levels were expressed as percentage of baseline, calculated as the ratio between Bb band intensity -either after formation in the presence of regulators or after the decay step- and the corresponding baseline Bb band formed in the absence of regulators x100. Data are shown as mean of n = 2 independent experiments for P5 (A) and P1 (B).
